# Exciting Opportunities in Nuclear Medicine Imaging and Therapy

**DOI:** 10.3390/jcm8111944

**Published:** 2019-11-12

**Authors:** Constantin Lapa

**Affiliations:** Department of Nuclear Medicine, University Hospital Augsburg, Stenglinstr. 2, 86156 Augsburg, Germany; Constantin.Lapa@uk-augsburg.de; Tel.: +49-821-400-2050

**Keywords:** molecular imaging, theranostics, prostate-specific membrane antigen, somatostatin receptor, molecular imaging reporting and data systems

## Abstract

Nuclear medicine has experienced a number of unprecedented developments in recent years. Above all, the concept of “theranostics”, the combination of a predictive biomarker with a therapeutic agent, has been a central part of this success. For example, a phase III randomized, controlled trial provided unequivocal evidence of the effectiveness of 177Lu-DOTATATE for treatment of neuroendocrine tumors, and there have been multiple reports of the benefits of prostate-specific membrane antigen targeted PET imaging and radio-ligand therapy in prostate cancer. Other new exciting theranostic applications include, among many others, C-X-C motif chemokine receptor 4, as well as cancer-associated fibroblasts. These can be specifically addressed by inhibitors of the fibroblast activation protein and represent a particularly promising target for nuclear medicine theranostics. This Special Issue presents some of the most recent advances in the field of nuclear medicine.

During the last few years, we have witnessed unprecedented advances in the field of nuclear medicine. One of the main driving forces is the so-called theranostic concept that combines the use of a diagnostic biomarker with a therapeutic option. Although the general idea of using a radioactive compound for diagnostic target-expression confirmation and subsequent radionuclide therapy has been established in nuclear medicine for more than 70 years (in terms of radioiodine therapy for thyroid cancer), recent developments have significantly broadened the scope of radionuclide imaging and therapies that now extends to neuroendocrine tumors [1], prostate cancer [2], or hematologic malignancies [3]. In addition to theranostics, other promising activities include efforts towards improved image reconstruction algorithms, standardized reporting, or the use of artificial intelligence [4,5,6,7].

Against the backdrop of these exciting developments, this Special Issue of the *Journal of Clinical Medicine* aims to provide an overview of the different facets of nuclear medicine. Starting with the potential relationship between malignancy-related bone marrow activation and prognosis [8], the Special Issue then features the value of nuclear imaging in inflammatory conditions such as Sjögren’s syndrome or sarcoidosis [9,10]. The next article addresses strategies to optimize reconstruction methods for dosimetry [11], whereas the last original contribution focuses on the association between diffuse thyroid uptake in 18F-FDG PET and the risk of dysfunction [12].

The second section consists of review articles and also provides an overview of various topics. In view of the recent Nobel Prize in Medicine, the first article on the role of PET-based hypoxia imaging in glioma treatment may be of particular interest [13]. Next, Werner and colleagues give an update on the current status of molecular imaging reporting and data systems for theranostic radiotracers, a very important step for future structured reporting, especially in the light of the growing use of somatostatin receptor- or prostate-specific membrane antigen-directed imaging [14]. The Special Issue concludes with an intriguing article on specific bacteria imaging [15].

In conclusion, I believe that this issue is as diverse and varied as the evolving field of nuclear medicine. At the moment, there is a plenitude of exciting innovation on the verge of or already in clinical application. I am convinced the future of our field seems promisingly bright and that there are unique opportunities to grow. I sincerely hope that this Special Issue helps to convey some of the enthusiasm and excitement and that you also enjoy exploring the different advances in nuclear medicine.
